# Effects of cognitively engaging physical activity on executive function, mental health, and physical fitness in school-age children: a cluster-randomized controlled trial

**DOI:** 10.1186/s40359-026-04339-2

**Published:** 2026-03-30

**Authors:** Yifan Xu, Kai Qi, Xiaoxiao Dong, Qi Xu, Shimeng Wang, Dandan Chen, Robert Schinke, Aiguo Chen

**Affiliations:** 1 Gdansk University of Physical Education and Sport, Gdansk, 80-336 Poland; 2 Nanjing Sport Institute, Nanjing, 210014 China; 3 Nantong University, Nantong, 226019 Jiangsu China; 4 Ningbo University, Ningbo, 315211 Zhejiang China; 5 School of Kinesiology and Health Sciences, Laurentian University, Sudbury, ON P3E 2C6 Canada

**Keywords:** Physical activity, School-aged children, Executive function, Resilience, Emotion regulation, Physical fitness

## Abstract

**Background:**

Over 80% of school-aged children worldwide engage in insufficient physical activity, compromising their executive function development during a critical neuroplasticity window. Cognitively Engaging Physical Activity (CEPA), which integrates cognitive challenges into physical exercise, may offer superior benefits compared to traditional physical education curriculum (TPEC), yet comprehensive evidence across cognitive, psychological, and physical domains remains limited. This study examined the differential effects of CEPA and TPEC on executive function (EF), mental health, and physical fitness in school-aged children.

**Methods:**

A total of 96 school-aged children were randomly assigned to either the CEPA group (*n* = 48, age = 9.31 ± 0.47, 54% boys) or the TPEC group (*n* = 48, age = 9.40 ± 0.49, 52% boys). The intervention lasted for 11 weeks, with sessions held three times per week for 45 min each. Pre- and post-intervention assessments included overall executive function (quantified by composite EF score), mental health (general mental health, resilience, emotion regulation), and physical fitness (speed, endurance, explosive power, flexibility, agility and coordination). A mixed-design ANOVA was conducted, and effect sizes were reported as partial eta squared (η²_*p*_).

**Results:**

The CEPA group demonstrated superior improvements (time*group interaction effects) in executive function (η²_*p*_ = 0.634), resilience (η²_*p*_ = 0.042), emotion regulation( η²_*p*_ = 0.081), speed (η²_*p*_ = 0.048), explosive power (η²_*p*_ = 0.041), endurance (η²_*p*_ = 0.627), and agility (η²_*p*_ = 0.181) compared to controls (all *p* < 0.05). No significant time*group effects were observed for overall mental health or flexibility. Girls showed greater speed improvements than boys in the CEPA condition (interaction *p* = 0.039).

**Conclusions:**

Within the studied cohort, the 11-week CEPA intervention was more effective than the TPEC in improving EF, The 11-week CEPA intervention was more effective than the TPEC in improving EF, resilience, emotion regulation, speed, endurance, and agility-coordination, with gender differences evident in speed performance. As a low-cost and scalable approach, CEPA provides empirical support for school-based health promotion and offers a promising public health strategy for enhancing children’s physical and mental development.

**Trial registration:**

This study was retrospectively registered at ChiCTR (Identifier ChiCTR2500101607) on April 27, 2025.

## Introduction

Childhood is a critical period for cognitive, emotional, and physical development. Executive function (EF), the cognitive processes that enable goal-directed behavior [1, 2], underlies academic success and social adaptation during these formative years [3]. Yet over 80% of school-aged children worldwide engage in insufficient physical activity [4], with children aged 6–9 spending 27.4% to 71.3% of their waking hours in sedentary behaviors [5–7]. This inactivity increases obesity risk, cardiovascular disease vulnerability, and compromises cognitive development [8–10]. Concurrently, academic pressure and social challenges intensify emotional vulnerability, manifesting as diminished resilience and emotion regulation difficulties [11–13]. Thus, intervention strategies that simultaneously address cognitive, emotional, and physical health are urgently needed in education and public health.

These developmental challenges are interconnected: EF, mental health, and physical fitness form a mutually reinforcing system [14–16]. During the critical period of 6–12 years, enhanced prefrontal cortex plasticity supports EF development and emotion regulation [17, 18]. This neuroplasticity makes physical activity particularly effective for enhancing EF during these years, as movement stimulates prefrontal activation and improves cognitive control [19]. Resilience (the capacity to adapt to stress) and emotion regulation (the ability to modulate emotional responses) are essential for academic and social demands [20, 21]. Physical fitness components including speed, endurance, explosive power, flexibility, agility, and coordination provide the physiological foundation for cognitive and emotional development [22, 23]. Research further demonstrates bidirectional relationships whereby physical fitness supports cognitive development and buffers psychological distress, while enhanced executive function facilitates emotion regulation [24, 25]. Importantly, interventions targeting single domains often show limited transfer effects [26, 27], underscores the need for intervention approaches that simultaneously target EF, mental health, and physical fitness.

Cognitively Engaging Physical Activity (CEPA) has emerged as a promising approach to address these interconnected developmental needs. Research has progressively demonstrated that cognitively demanding physical activities yield superior cognitive benefits compared to traditional exercise approaches [28]. Early work by Budde et al. showed that coordinative exercise acutely improves attentional performance [29], while subsequent studies have extended these findings to chronic interventions [30]. CEPA builds on this foundation by systematically integrating cognitive tasks into physical activity through elements such as decision-making, strategic adjustment, and attentional control [31, 32]. This approach is theoretically grounded in complementary frameworks. The Dual-Task training theory [33] posits that simultaneous physical-cognitive demands enhance resource allocation more effectively than single-task activities. This aligns with the Cognitive Stimulation Hypothesis [34, 35], which emphasizes that exercise quality, specifically the cognitive enrichment within movement, matters more than quantity for executive function enhancement [28, 35]. Cognitively demanding activities requiring continuous decision-making and environmental adaptation stimulate greater neural activation than repetitive, automated movements [29, 36]. These concurrent cognitive-motor demands activate prefrontal regions involved in both executive control and emotion regulation [37, 38], potentially yielding broader developmental benefits. Compared with TPEC, which emphasizes repetitive practice and basic motor skills, CEPA stimulates greater neural activation through its cognitive challenges [39]. Research indicates that CEPA’s diverse activity formats and novelty increase children’s intrinsic motivation and sustained engagement [40].

Previous studies have provided preliminary evidence for the positive effects of CEPA on EF, though findings remain inconsistent. While recent meta-analyses report moderate to large effects on executive function [31], individual studies show considerable variation. Some find cognitive engagement crucial for EF improvements [41], others report no effect [42, 43] or even negative effects [44]. These inconsistencies likely stem from differences in intervention duration, intensity, and assessment methods. Notably, most studies focus solely on cognitive outcomes, neglecting the potential benefits for mental health and physical fitness. Furthermore, few investigations have employed comprehensive, long-duration protocols necessary to establish stable effects.

Beyond cognitive benefits, CEPA may offer unique advantages for children’s mental health. The cognitive challenges inherent in CEPA may enhance both resilience and emotion regulation through central executive network activation [45] and achievement experiences [46]. While traditional physical activity improves mood and reduces stress [25, 47], CEPA’s integration of cognitive and physical demands potentially amplifies these benefits beyond traditional physical activity alone [48]. However, empirical evidence for CEPA’s specific effects on mental health outcomes remains scarce, with most studies focusing exclusively on cognitive outcomes.

Physical fitness, the third crucial dimension, represents an often-overlooked benefit of CEPA. Physical fitness components including speed, endurance, explosive power, flexibility, agility, and coordination are fundamental to children’s motor development and daily functional capacity [49, 50]. TPEC often relies on repetitive drills that may diminish motivation and limit fitness gains [51]. In contrast, CEPA may enhance motor competence across multiple domains by simultaneously integrating cognitive challenges into physical movement tasks [52]. The cognitive demands require rapid decision-making and adaptive responses, potentially stimulating neuromuscular adaptations beyond those achieved through repetitive practice alone [53]. Yet CEPA research has overwhelmingly focused on EF, with minimal evidence regarding physical fitness outcomes, despite the clear theoretical rationale for such benefits.

Previous studies have provided preliminary evidence for CEPA’s positive effects, yet comprehensive evaluation across cognitive, mental health, and physical fitness domains remains limited. Given CEPA’s dual nature of integrating cognitive challenges into physical movement, its effects should theoretically extend beyond EF to encompass all three dimensions. Comprehensive assessment is therefore essential for capturing CEPA’s full intervention effects and identifying its unique advantages over traditional approaches [54]. This study examined CEPA’s impact on three key developmental dimensions in school-aged children. Such evidence is crucial for developing practical, evidence-based interventions that schools can implement to address multiple developmental needs simultaneously. We hypothesized that compared to TPEC, an 11-week CEPA intervention would significantly improve: (1) executive function performance, (2) resilience and emotion regulation capabilities, and (3) physical fitness indicators including speed, endurance, explosive power, flexibility, agility, and coordination. The findings may provide evidence-based guidance for developing comprehensive school-based interventions that promote children’s holistic development through cognitively engaging physical activities.

## Methods

### Design

This study followed CONSORT guidelines for cluster-randomized controlled trials and employed a two-stage cluster sampling approach [55]. The study was conducted from September 2024 to January 2025 at a single primary school in Yangzhou City, China. In the first stage, fourth-grade classes were invited to participate through school-wide announcements, with interested classes voluntarily enrolling in the recruitment pool (13 classes enrolled). In the second stage, two intact classes were randomly selected from these 13 volunteering classes using a computer-generated random number table and assigned to either the experimental condition (CEPA intervention group, *n* = 52 students) or the control condition (TPEC group, *n* = 52 students).

To minimize potential biases, several measures were implemented: (1) Allocation concealment: The informed consent described the study as “comparing different physical education teaching methods” without revealing specific intervention details. Neither students, parents, nor teachers knew which group was experimental or control until study completion. (2) Assessor blinding: All outcome assessors were blinded to group allocation throughout the study. (3) Implementation fidelity: Although the same teacher delivered both interventions to minimize instructor effects, strict protocols and researcher monitoring were employed to ensure adherence to assigned curricula and reduce potential contamination between groups. Additionally, we employed an active control condition (TPEC) rather than a passive control to isolate the specific effects of cognitive engagement while controlling for nonspecific treatment effects such as physical activity exposure and social interaction [56].

Baseline assessments were conducted in September 2024, followed by an 11-week intervention period, with post-intervention assessments completed in January 2025. Only participants who completed baseline and post-intervention assessments were included in the analyses. Outcome measures covered the following domains: EF, psychological indicators (mental health, resilience, and emotion regulation), and physical fitness (speed, strength, endurance, flexibility, agility, and coordination).

### Participants

A total of 104 students from two fourth-grade classes at a primary school in Yangzhou City, China were enrolled in this cluster-randomized trial. Through class-level randomization, one class (*n* = 52 students) was allocated to the experimental group and another class (*n* = 52 students) to the control group. During the intervention period, in the control group, two students dropped out due to school transfer and two students did not complete the full intervention. In the experimental group, 4 students did not complete the full intervention. The final sample comprised 96 students who completed all assessments: the experimental group (*n* = 48; 26 boys, 22 girls; mean age = 9.31 ± 0.47 years) and the control group (*n* = 48; 25 boys, 23 girls; mean age = 9.40 ± 0.49 years).

Cluster-level selection criteria: Fourth-grade classes with voluntary participation from all students and their legal guardians were eligible for inclusion in the recruitment pool.Individual-level inclusion criteria were as follows: (1) the ability to safely participate in regular physical education classes, confirmed by school health records and teacher reports; (2) no prior participation in structured cognitive-motor training programs or psychological interventions; (3) written informed consent provided by legal guardians and child assent obtained following age-appropriate explanations. Individual-level exclusion criteria included: (1) diagnosed psychological, neurological, or developmental disorders (e.g., ADHD, ASD, depression); (2) medical contraindications or physical conditions (e.g., recent injury, chronic illness) affecting safe physical activity; and (3) participation in other structured cognitive-motor training or psychological programs within the past three months. These criteria were intended to control for confounding variables that could bias the interpretation of intervention effects.

**Fig. 1:**
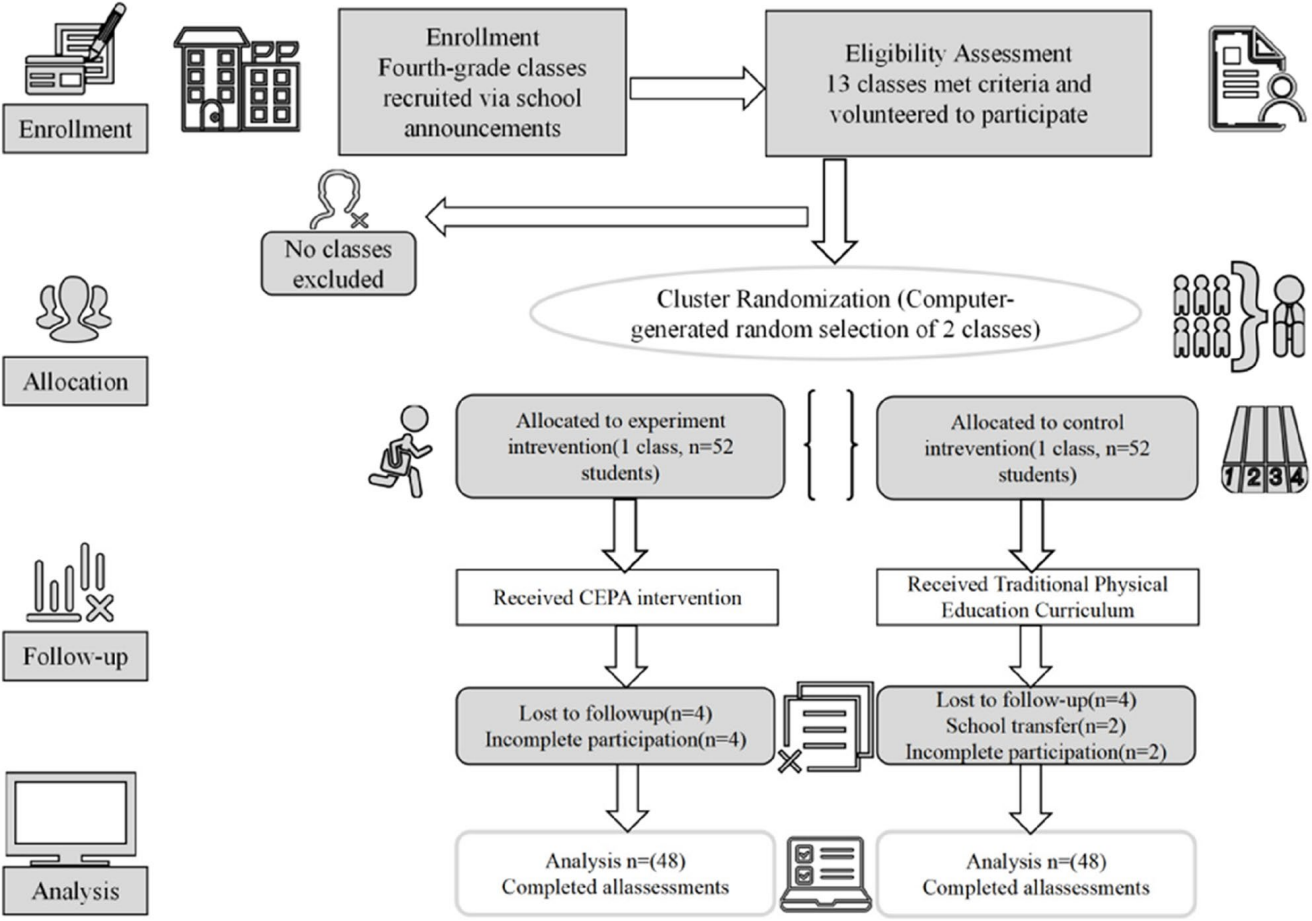
Flowchart of participant selection

Sample size calculation was conducted using G*Power 3.1.9.7 based on repeated-measures ANOVA [57]. With an effect size of 0.25 (moderate), a significance level of 0.05, and a power of 0.85, the required sample size was 76. To account for potential attrition, all students from the two selected classes were recruited (*N* = 104). During the intervention period, eight students were lost to follow-up (4 from each group), resulting in 48 students per group completing the study (retention rate: 92.3%). The flow of participants through the study is shown in Fig. 1.

During the intervention, the participants were explicitly instructed not to engage in any additional extracurricular physical or psychological training. Implementation fidelity and participant compliance were monitored through direct observation of all sessions by research team members, who verified protocol adherence and recorded attendance. Only the participants who completed both assessments and attended all intervention sessions were included in the final analysis.

The study was conducted in accordance with the ethical standards outlined in the Declaration of Helsinki and was approved by the Ethics Review Committee of Yangzhou University School of Medicine (Approval No. YXYLL-2023-129). Written informed consent was obtained from all legal guardians, and age-appropriate assent was obtained from all participating children.

### Procedure

All measurements were conducted during regular teaching hours over two weeks. Executive function assessments were conducted in a quiet computer room. Psychological questionnaires were administered in classrooms with trained researchers providing standardized instructions. Physical fitness measurements were conducted in the school gymnasium under controlled environmental conditions, between 9:00–11:00 a.m. to minimize circadian rhythm effects [58].

The assessment team consisted of trained sports professionals who underwent standardized training on measurement protocols, data recording, and inter-rater reliability before data collection. Daily briefings ensured consistency across assessors throughout the study.

Before formal testing, all participants received standardized instructions followed by practice sessions to ensure task comprehension. Adequate rest periods (1–5 min) were provided between tests to minimize fatigue effects.

### Intervention

**Fig. 2:**
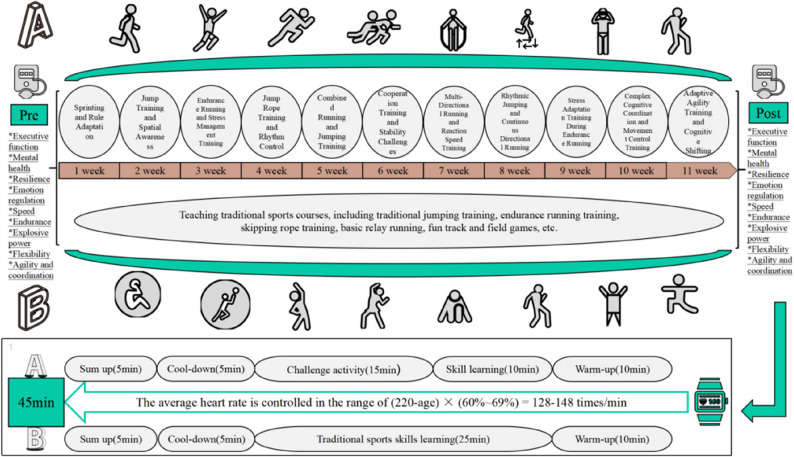
Schematic diagram of the 45-minute class structure for CEPA (**A**) and control (**B**) groups. CEPA sessions included “Challenge activities” (cognitively demanding tasks) and “Skill learning” (movement practice within cognitive contexts). Control sessions featured “Traditional sports skills learning” (conventional technique instruction without cognitive training)

The intervention lasted for 11 weeks, with sessions conducted three times per week, each lasting approximately 45 min. Exercise intensity was maintained at a moderate level throughout, monitored using Polar M430 with target heart rate of 128–148 beats per minute. This target heart rate zone was determined based on the Heart Rate Reserve (HRR) principle, corresponding to moderate intensity (50–70% HRR) for children aged 9–10 years, ensuring appropriate intensity control across both groups [59, 60]. Each session followed a standard structure: 10-minute warm-up, 25-minute main activities, 5-minute reflective summary, and 5-minute cool-down. The specific classroom procedures are illustrated in Fig. 2.

Each CEPA session comprised two main components: (1) “Challenge activities” consisted of cognitively demanding physical tasks requiring problem-solving and adaptation, such as relay races with decision-making requirements and team games with changing rules; (2) “Skill learning” involved the introduction and practice of new movement patterns within cognitive contexts. In contrast, the TPEC’s sessions focused on “traditional sports skills learning” following the national Physical Education and Health curriculum standards, emphasizing conventional technique instruction and repetitive motor skill practice without cognitive training elements. Detailed weekly lesson plans for both groups are provided in Supplementary Material 1.Both two groups were taught by the same trained physical education teacher to control for instructor effects.

### Measures

#### Executive function

Executive function was assessed using a computerized neuropsychological test battery specifically developed for Chinese children by Professor Aiguo Chen [61], which has been validated in previous studies with Chinese children [14]. The battery comprised three tasks measuring core EF components: the Flanker task for inhibitory control (48 test trials), the 1-back task for working memory (25 test trials), and the More-odd shifting task for cognitive flexibility (32 switch trials). Inhibition was indexed by interference cost (incongruent RT minus congruent RT), cognitive flexibility by switch cost (switch RT minus non-switch RT), and working memory by mean RT on the 1-back task. Following established scoring protocols for this battery [62, 63], a composite EF score (sum of the three indices, in milliseconds) was used as the primary outcome, with lower scores indicating better performance.

All tasks were administered using E-Prime software by trained examiners. To ensure task comprehension, participants were required to achieve ≥ 60% accuracy in practice trials before formal testing. Those not meeting this criterion received additional instruction until meeting this threshold. Response times < 200ms or > 3000ms were excluded as outliers, and only correct trials were included in analysis.

#### Mental health

##### Mental health test

The Mental Health Test (MHT) was adapted from the Anxiety Tendency Test and standardized for Chinese populations [64]. The Chinese version has been widely validated among Chinese children [65–67]. The MHT consists of 100 binary items covering eight dimensions (learning anxiety, social anxiety, loneliness, self-blame, sensitivity, physical symptoms, fear, and impulsiveness), with total scores ≥ 65 indicating serious mental health problems. Consistent with our focus on overall mental health, we used the total score.

##### Resilience

The Resilience Scale for Chinese Adolescents (RSCA), developed by Xueqin Hu specifically for Chinese children [68], is highly suitable for assessing resilience in this population. It has demonstrated good reliability and validity [69, 70]. The 27-item scale uses a 5-point Likert scale (1 = strongly disagree to 5 = strongly agree). Reverse-scored items were recoded before computing the total score, with higher scores indicating greater levels of resilience.

##### Emotion regulation

The Emotion Regulation Questionnaire for Children and Adolescents (ERQ-CA) was used in this study. The original English version was developed by Gullone and Taffe based on the ERQ [71], and Chen Liang adapted the Chinese version [72]. The scale has demonstrated good reliability and validity among Chinese primary school students [73], ensuring its applicability in this cultural setting. The ERQ-CA consists of 10 items and a 5-point Likert scale ranging from 1 (completely disagree) to 5 (completely agree). Higher total scores indicate more frequent use of the respective emotion regulation strategy.

#### Physical fitness

##### Speed

The 50-meter sprint is a commonly used indicator for assessing speed in children [74]. Participants stood at a standard starting line and began running in response to the command “run.” Time (s) were recorded, and the measurement ended when the participants’ chest crossed the vertical plane of the finish line. Each participant completed two trials, and the better result was recorded. The mean within-participant coefficient of variation across trials was 3.1%.

##### Endurance

The one-minute rope skipping test (measuring jumps per minute) has been shown to be a valid indicator of endurance in typically developing children [75]. Before testing, students were assisted in adjusting the rope to an appropriate length. A timekeeper recorded the number of successful jumps completed within one minute. The movement required alternate foot landings and a full rope pass beneath the body. Missed or paused jumps were not counted. Each participant completed one trial, and the total number of successful jumps was recorded.

##### Explosive power

The standing long jump is a valid measure of lower limb explosive power and overall muscular fitness in children [76]. Participants jumped from a standing position, and the longest distance was recorded. Arm swing and forward lean were allowed; backward steps were not. Distance was measured to the nearest centimeter from the starting line to the closest landing point. Each participant completed two trials; the better result was recorded. The average within-participant coefficient of variation was 3.8%.

##### Flexibility

The sit-and-reach test is a widely used measure of lower back and hamstring flexibility [77]. Participants placed their feet flat against the vertical board with legs straight and pushed the cursor forward with extended arms until maximum reach. Each participant completed two trials, with the better result (cm) recorded. The average within-participant coefficient of variation across trials was 1.6%.

##### Agility and coordination

The cross-quadrant jump is a simple and reliable test of agility and coordination in children [78]. Two 1-meter lines were drawn perpendicularly on the floor, dividing the surface into four labeled quadrants (1–4). Participants began in quadrant 1 with feet together and jumped in the sequence"1→2→3→4→1”, returning to the center after each jump. Each jump had to be completed with both feet off the ground, without stepping on lines or repeating quadrants. Invalid jumps were excluded from the total. The time (in seconds) to complete 10 jumps was recorded.

### Statistical analysis

Descriptive statistics were presented as means and standard deviations, and the Shapiro-Wilk test and Levene test were used to assess the normality and homogeneity of the data, respectively. It indicated normality (*p* > 0.05) and homogeneity (*p* > 0.05). Independent t-tests and Cohen’s d effect size tests were performed to compare baseline levels between groups. A mixed ANOVA (times * group) was used to examine the interaction between factors, combined with partial eta squared (η²_*p*_) to measure effect sizes, based on established thresholds, interpreted as > 0.01 (small), > 0.06 (medium), and > 0.14 (large) [79]. Given that only two clusters were available (one per condition), multilevel modeling was not feasible due to insufficient units for reliable between-cluster variance estimation [80, 81]. Mixed ANOVA was therefore used, and intra-class correlations (ICCs) were calculated at baseline to evaluate potential clustering effects.The Bonferroni test was used for post-hoc comparisons.For all time × group interaction effects addressing our primary intervention hypothesis, 95% confidence intervals for partial eta squared (η²p) were calculated using non-central F distribution methods [82, 83]. All statistical analyses were performed with the use of SPSS29.0, with a predetermined significance level of *p* < 0.05. Figures were created using Origin 2021 (OriginLab Corporation, Northampton, MA, USA).

## Results

### Baseline assessments

A total of 96 participants (48 in the CEPA group and 48 in the TPEC group) who completed all tests were included in the final analysis. The mean and standard deviation of the baseline indicators—age, EF, mental health (including resilience and emotion regulation), and physical fitness (speed, endurance, explosive power, flexibility, agility and coordination)—for both groups are presented in Table 1. There were no significant differences between the CEPA and TPEC groups in terms of gender ratio (*p* = 0.840), age (*p* = 0.361), EF (*p* = 0.870), mental health (*p* = 0.591), resilience (*p* = 0.742), or emotion regulation (*p* = 0.340). Similarly, no significant differences were found between the two groups in physical fitness indicators, including cross-quadrant jump (*p* = 0.783), standing long jump (*p* = 0.640), 50-metre run (*p* = 0.315), seated forward bending (*p* = 0.590), and jumps per minute (*p* = 0.472). These findings indicate that both groups demonstrated baseline equivalence prior to the intervention. Intra-class correlations (ICCs) for all baseline outcome measures were negligible (range: 0–0.001), with design effects ranging from 1.00 to 1.047, indicating minimal clustering effects.

**Table 1:** Characteristics of the study participants

Measure	Experimental group (*n* = 48)	Control group (*n* = 48)	t	*p*	d
Boys	26	25	0.20	0.840	0.04
Girls	22	23	-0.20	0.840	-0.04
Age (years)	9.31 ± 0.47	9.40 ± 0.49	-0.92	0.361	-0.19
Executive function (ms)	1197.79 ± 185.74	1204.08 ± 189.64	-0.16	0.870	-0.03
Mental Health	29.92 ± 16.32	31.85 ± 18.77	-0.54	0.591	-0.11
Resilience	94.83 ± 18.02	96.06 ± 18.42	-0.33	0.742	-0.07
Emotion regulation	26.06 ± 5.11	26.96 ± 3.96	-0.96	0.340	-0.20
50-m sprint (s)	10.69 ± 0.95	10.90 ± 1.06	-1.01	0.315	-0.21
Jumps per minute (number)	93.40 ± 29.15	97.58 ± 27.58	-0.72	0.472	-0.15
Standing Long Jump (cm)	125.27 ± 11.62	124.19 ± 10.99	0.47	0.640	0.10
sit-and-reach test (cm)	6.29 ± 7.74	7.15 ± 7.97	-0.54	0.590	-0.11
cross-quadrant jump (s)	46.53 ± 12.41	45.81 ± 13.18	0.28	0.783	0.06

Data were expressed as mean ± standard deviation (M ± SD); *p* value indicates the significance of the difference between groups; d is Cohen’s d effect size; Executive function measured by composite EF score (sum of interference cost, mean RT, and switch cost)

### Descriptive statistics, effect sizes, and intervention effects

This section presents the intervention effects across all outcome measures. Descriptive statistics and within-group effect sizes by gender are detailed in Tables 2 and 3. Group-level descriptive statistics are provided in Supplementary Table S1. Mixed ANOVA results are summarized in Table 4.

**Table 2:** Descriptive statistics of boys and girls subjects before and after the intervention

	Group	Pre-test(Mean ± SD)			Post-test (Mean ± SD)			Change		
		Overall	Boys	Girls	Overall	Boys	Girls	Overall	Boys	Girls
Executive Function(ms)	EG	1197.79 ± 185.74	1192.66 ± 185.45	1203.85 ± 190.26	1010.26 ± 128.55	1001.28 ± 121.11	1020.88 ± 138.94	-15.66%; d= -1.17 *p* < 0.001	-16.05%; d=-1.22 *p* < 0.001	-15.20%; d= -1.08 *p* < 0.001
	CG	1204.08 ± 189.64	1255.00 ± 179.02	1148.73 ± 189.00	1180.01 ± 131.55	1214.72 ± 123.90	1142.28 ± 131.77	-2.00%; d= -0.15 *p* = 0.012	-3.21%; d= -0.26 *p* = 0.002	-0.56%; d= -0.04 *p* = 0.627
Mental Health	EG	29.92 ± 16.32	28.65 ± 16.39	31.41 ± 16.49	26.50 ± 9.70	23.92 ± 9.45	29.55 ± 9.29	-11.43%; d= -0.25 *p* = 0.178	-16.51%; d= -0.36 *p* = 0.153	-5.92%; d= -0.14 *p* = 0.603
	CG	31.85 ± 18.77	33.60 ± 20.35	29.96 ± 17.15	30.60 ± 16.73	33.44 ± 15.99	27.52 ± 17.31	-3.93%; d= -0.07 *p* = 0.593	-0.48%; d= -0.01 *p* = 0.962	-8.14%; d= -0.14 *p* = 0.488
Resilience	EG	94.83 ± 18.02	96.65 ± 17.32	92.68 ± 18.98	105.00 ± 21.94	109.08 ± 21.59	100.18 ± 21.86	10.72%; d = 0.51 *p* < 0.001	12.86%; d = 0.64 *p* < 0.001	8.09%; d = 0.37 *p* = 0.058
	CG	96.06 ± 18.42	93.08 ± 17.84	99.30 ± 18.88	98.50 ± 19.13	95.84 ± 16.97	101.39 ± 21.23	2.54%; d = 0.13 *p* = 0.363	2.97% ; d = 0.16 *p* = 0.454	2.10%; d = 0.10 *p* = 0.587
Emotion Regulation	EG	26.06 ± 5.11	25.50 ± 3.92	26.73 ± 6.27	30.15 ± 3.67	29.46 ± 3.27	30.95 ± 4.03	15.69%; d = 0.92 *p* < 0.001	15.53%; d = 1.09 *p* < 0.001	15.79%; d = 0.80 *p* < 0.001
	CG	26.96 ± 3.96	26.56 ± 3.78	27.39 ± 4.20	28.50 ± 4.55	27.88 ± 4.61	29.17 ± 4.48	5.71%; d = 0.36 *p* = 0.016	4.97% ; d = 0.31 *p* = 0.135	6.50%; d = 0.41 *p* = 0.054

*EG* Experimental group, *CG* Control group

For executive function, negative Cohen’s d values indicate improvement (shorter reaction times represent better performance); Executive function measured by composite EF score (sum of interference cost, mean RT, and switch cost)

**Table 3:** Descriptive statistics of boys and girls subjects before and after physical fitness

	Group	Pre-test(Mean ± SD)			Post-test (Mean ± SD)			Change		
		Overall	Boys	Girls	Overall	Boys	Girls	Overall	Boys	Girls
50-meter Sprint(s)	EG	10.69 ± 0.95	10.29 ± 0.71	11.16 ± 0.99	9.85 ± 0.31	9.92 ± 0.26	9.77 ± 0.35	7.84%; d = 1.14 *p* < 0.001	3.60%; d = 0.68 *p* = 0.124	12.46%; d = 1.84 *p* < 0.001
	CG	10.90 ± 1.06	10.84 ± 1.11	10.97 ± 1.02	10.55 ± 0.66	10.48 ± 0.63	10.63 ± 0.69	3.21%; d = 0.39 *p* = 0.050	3.32%; d = 0.40 *p* = 0.143	3.10%; d = 0.40 *p* = 0.184
Jumps per minute (number)	EG	93.40 ± 29.15	93.15 ± 32.94	93.68 ± 24.70	117.48 ± 31.87	115.77 ± 34.32	119.50 ± 29.38	25.79%; d = 0.79 *p* < 0.001	24.28%; d = 0.68 *p* < 0.001	27.56%; d = 0.96 *p* < 0.001
	CG	97.58 ± 27.58	99.76 ± 26.75	95.22 ± 28.87	103.81 ± 25.95	106.48 ± 26.22	100.91 ± 25.93	6.38%; d = 0.23 *p* < 0.001	6.72%; d = 0.26 *p* < 0.001	5.97%; d = 0.21 *p* < 0.001
Standing Long Jump(cm)	EG	125.27 ± 11.63	124.88 ± 11.80	125.73 ± 11.68	139.69 ± 25.79	150.77 ± 20.36	126.59 ± 25.76	11.51%; d = 0.71 *p* < 0.001	20.73%; d = 1.57 *p* < 0.001	0.68%; d = 0.04 *p* = 0.872
	CG	124.19 ± 10.99	125.84 ± 9.89	122.39 ± 12.03	127.50 ± 18.41	132.49 ± 17.44	122.08 ± 18.24	2.67%; d = 0.22 *p* = 0.384	5.28%; d = 0.55 *p* = 0.188	-0.25%; d= -0.03 *p* = 0.952
sit-and-reach test (cm)	EG	6.29 ± 7.74	3.72 ± 5.94	9.31 ± 8.62	6.34 ± 7.83	2.40 ± 5.68	11.00 ± 7.53	0.95%; d = 0.01 *p* = 0.853	-35.48%; d= -0.23 *p* = 0.326	18.15%; d = 0.21 *p* = 0.249
	CG	7.15 ± 7.97	3.33 ± 6.68	11.30 ± 7.24	7.12 ± 5.11	4.78 ± 4.71	9.66 ± 4.31	-0.43%; d = 0.00 *p* = 0.918	43.54%; d = 0.25 *p* = 0.292	-14.51%; d= -0.27 *p* = 0.249
cross-quadrant jump (s)	EG	46.53 ± 12.41	43.58 ± 11.84	50.03 ± 12.43	30.79 ± 6.28	27.22 ± 5.10	35.01 ± 4.79	33.84%; d = 1.62 *p* < 0.001	37.52%; d = 1.80 *p* < 0.001	30.04%; d = 1.57 *p* < 0.001
	CG	45.81 ± 13.18	46.25 ± 13.71	45.34 ± 12.88	42.63 ± 4.61	41.81 ± 5.03	43.52 ± 4.02	6.94%; d = 0.31 *p* = 0.115	9.60%; d = 0.42 *p* = 0.107	4.01%; d = 0.19 *p* = 0.522

*EG* Experimental group, *CG* Control group

For time-based measures (50-m sprint and cross-quadrant jump), negative Cohen’s d values indicate improvement (shorter times represent better performance); for other measures, positive values indicate improvement

**Table 4:** Summary of Statistical Analyses for All Outcome Measures

Outcome Variable	Time	Group	Time × Group	Gender × Time × Group
Executive Function (ms)	F = 263.64 *p*<0.001^***^ η²_*p=*_0.741	F = 7.13 *p =* 0.009 η²_*p=*_0.072	F = 159.58 *p*<0.001^***^ η²_*p=*_0.634 95% CI [0.51, 0.71]	F = 0.96 *p*=0.329 η²_*p=*_0.010
Mental Health Total	F = 1.80 *p =* 0.183 η²_*p=*_0.019	F = 1.02 *p =* 0.316 η²_*p=*_0.011	F = 0.34 *p*=0.561 η²_*p=*_0.004 95% CI [0.00, 0.06]	F = 0.56 *p*=0.455 η²_*p=*_0.006
Resilience	F = 10.88 *p =* 0.001 η²_*p=*_0.106	F = 0.41 *p =* 0.522 η²_*p=*_0.004	F = 4.03 *p*=0.048^*^ η²_*p=*_0.042 95% CI [0.00, 0.14]	F = 0.32 *p*=0.573 η²_*p=*_0.003
Emotion Regulation	F = 39.72 *p*<0.001^***^ η²_*p=*_0.302	F = 0.28 *p =* 0.597 η²_*p=*_0.003	F = 8.06 *p*=0.006^**^ η²_*p=*_0.081 95% CI [0.01, 0.20]	F = 0.012 *p*=0.913 η²_*p=*_0.000
Speed(50-m sprint)	F = 24.51 *p*<0.001^***^ η²_*p=*_0.210	F = 20.55 *p*<0.001^***^ η²_*p=*_0.183	F = 4.59 *p*=0.035^*^ η²_*p=*_0.048 95% CI [0.00, 0.15]	F = 4.40 *p*=0.039^*^ η²_*p=*_0.046
Endurance(jumps per minute)	F = 442.06 *p*<0.001^***^ η²_*p=*_0.828	F = 0.70 *p =* 0.404 η²_*p=*_0.008.	F = 154.89 *p*<0.001^***^ η²_*p=*_0.627 95% CI [0.50, 0.71]	F = 2.13 *p*=0.148 η²_*p=*_0.023
Explosive Power(standing long jump)	F = 10.42 *p =* 0.002 η²_*p=*_0.102	F = 7.94 *p =* 0.006 η²_*p=*_0.079	F = 4.02 *p*=0.048^*^ η²_*p=*_0.041 95% CI [0.00, 0.14]	F = 3.10 *p*=0.082 η²_*p=*_0.033
Flexibility(sit-and-reach)	F = 0 *p =* 0.953 η²_*p=*_0	F = 0.35 *p =* 0.557 η²_*p=*_0.004	F = 0.04 *p*=0.838 η²_*p*_<0.001 95% CI [0.00, 0.01]	F = 4.79 *p*=0.031^*^ η²_*p=*_0.049
Agility(cross-quadrant jump)	F = 45.67 *p*<0.001^***^ η²_*p=*_0.332	F = 14.41 *p*<0.001^***^ η²_*p=*_0.135	F = 20.35 *p*<0.001^***^ η²_*p=*_0.181 95% CI [0.06, 0.31]	F = 0.05 *p*=0.822 η²_*p=*_0.001

#### Executive function

**Fig. 3:**
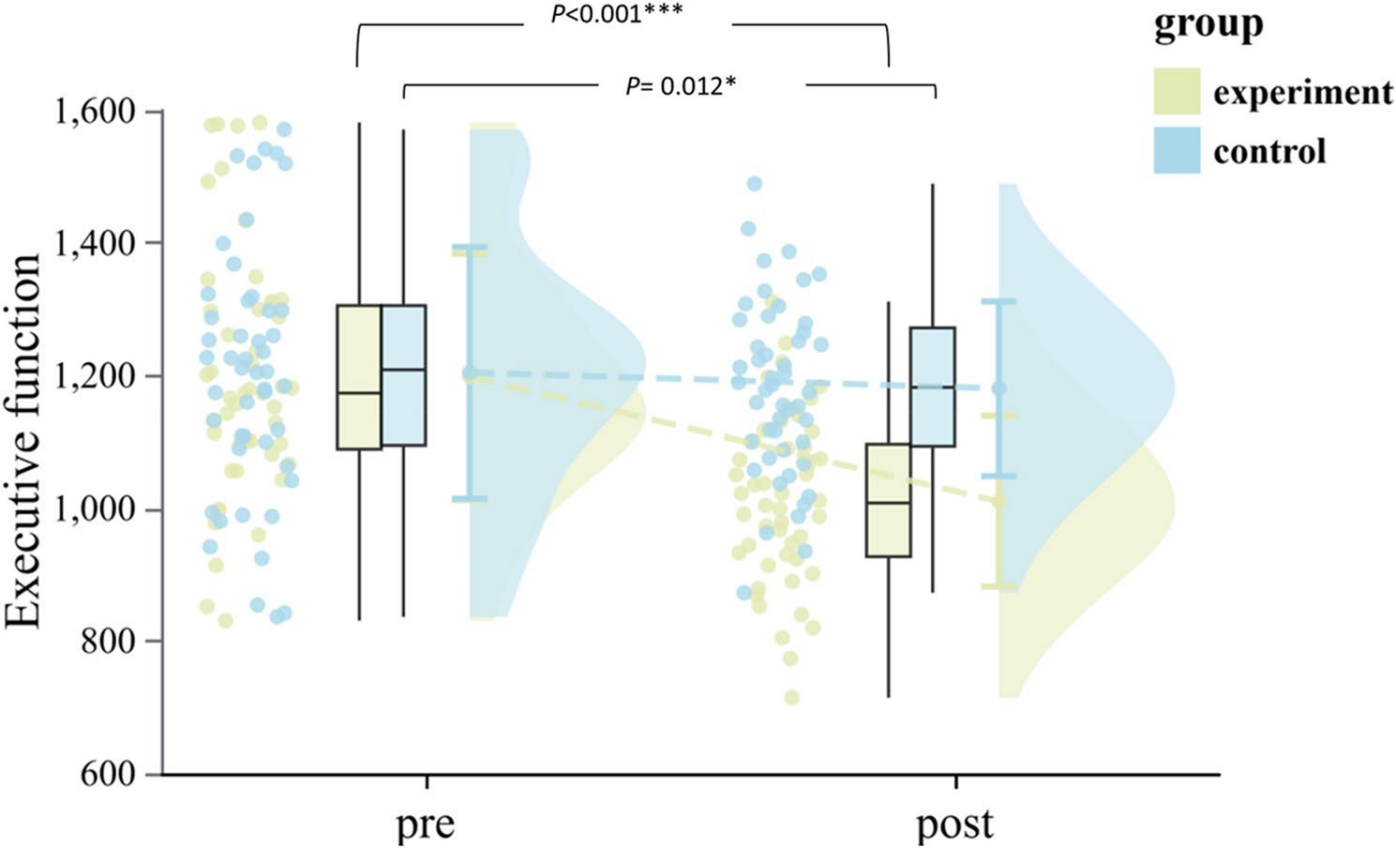
Within-group variation in EF in each group (pre vs. post). Y-axis represents composite EF score in milliseconds (ms). Experimental group: yellow, Control group: blue, ^*^p ≤ 0.05,^***^p < 0.001

The time × group interaction revealed substantial improvements favoring CEPA (F(1,92) = 159.58, *p* < 0.001, η²_*p*_ = 0.634, 95% CI [0.51, 0.71]; see Table 4). Simple effects analysis showed significant improvement in the CEPA group (*p* < 0.001) with composite EF scores decreasing 15.66% (from 1197.79 ± 185.74 to 1010.26 ± 128.55 ms), while controls showed minimal change of 2.00% (from 1204.08 ± 189.64 to 1180.01 ± 131.55 ms, *p* = 0.012). Gender did not moderate these effects (*p* = 0.329), with large effect sizes for both boys (d = 1.22) and girls (d = 1.08) in the CEPA group (see Table 2; Fig. 3).Task-level analyses confirmed significant improvements across all three EF components (see Supplementary Table S2).

#### Mental health

**Fig. 4:**
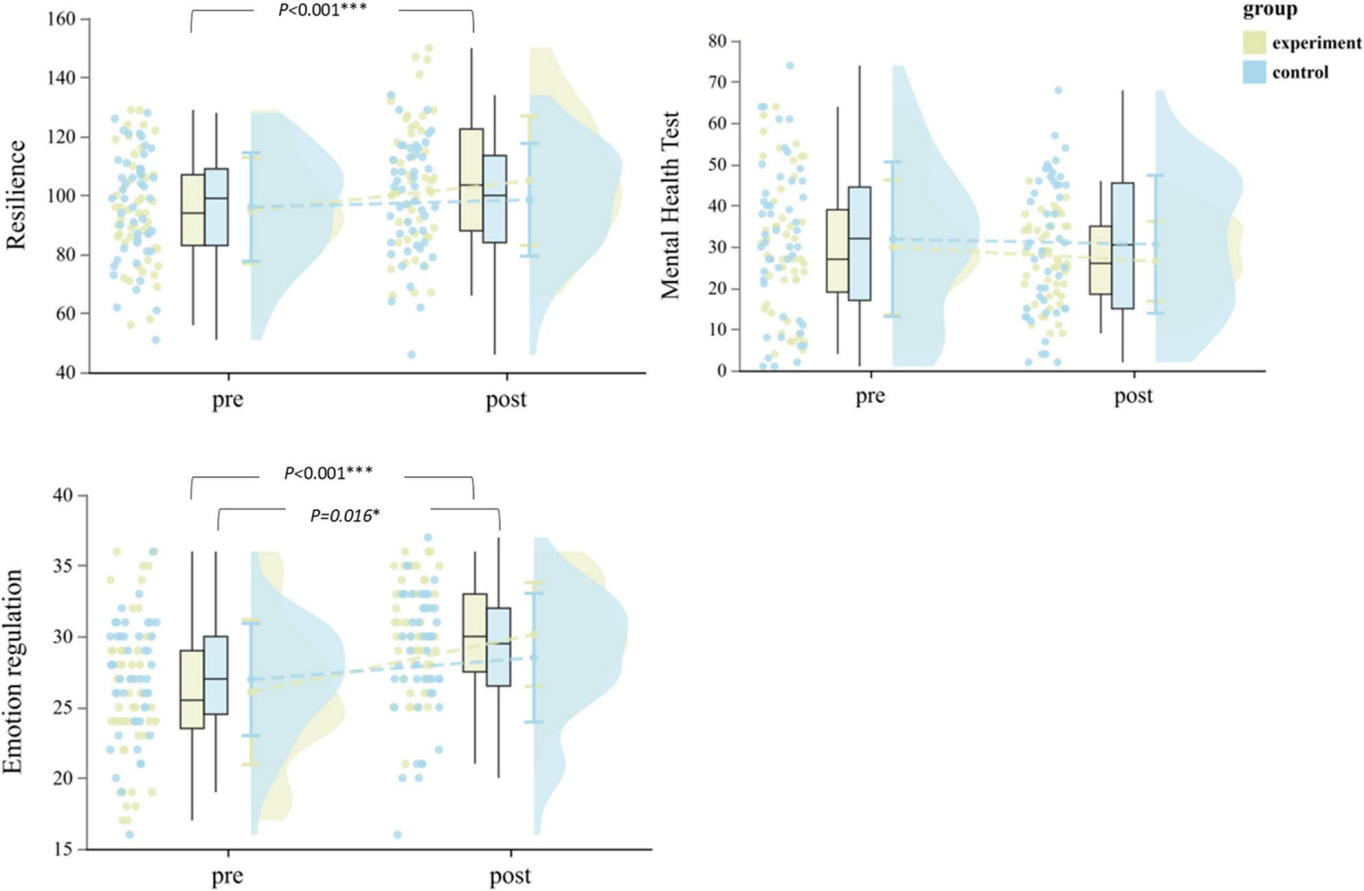
Within-group differences in mental health across groups. Experimental group: yellow, Control group: blue. ^*^p ≤ 0.05, ^***^p < 0.001

Overall mental health scores showed no significant intervention effects (time × group: *p* = 0.561; Table 4). Resilience demonstrated a significant time ×group interaction (F(1,92) = 4.03, *p* = 0.048, η²_*p*_ = 0.042, 95% CI [0.00, 0.14]), with the CEPA group improving 10.72% (from 94.83 ± 18.02 to 105.00 ± 21.94, *p* < 0.001) while controls showed no change (*p* = 0.363). Emotion regulation showed stronger effects (F(1,92) = 8.06, *p* = 0.006, η²_*p*_ = 0.081, 95% CI [0.01, 0.20]), improving 15.69% in CEPA participants (from 26.06 ± 5.11 to 30.15 ± 3.67, *p* < 0.001) versus 5.71% in controls (from 26.96 ± 3.96 to 28.50 ± 4.55, *p* = 0.016). Gender did not moderate psychological outcomes (all *p* > 0.45), with moderate to large effect sizes across both boys and girls (Table 2; Fig. 4).

#### Physical fitness

**Fig. 5:**
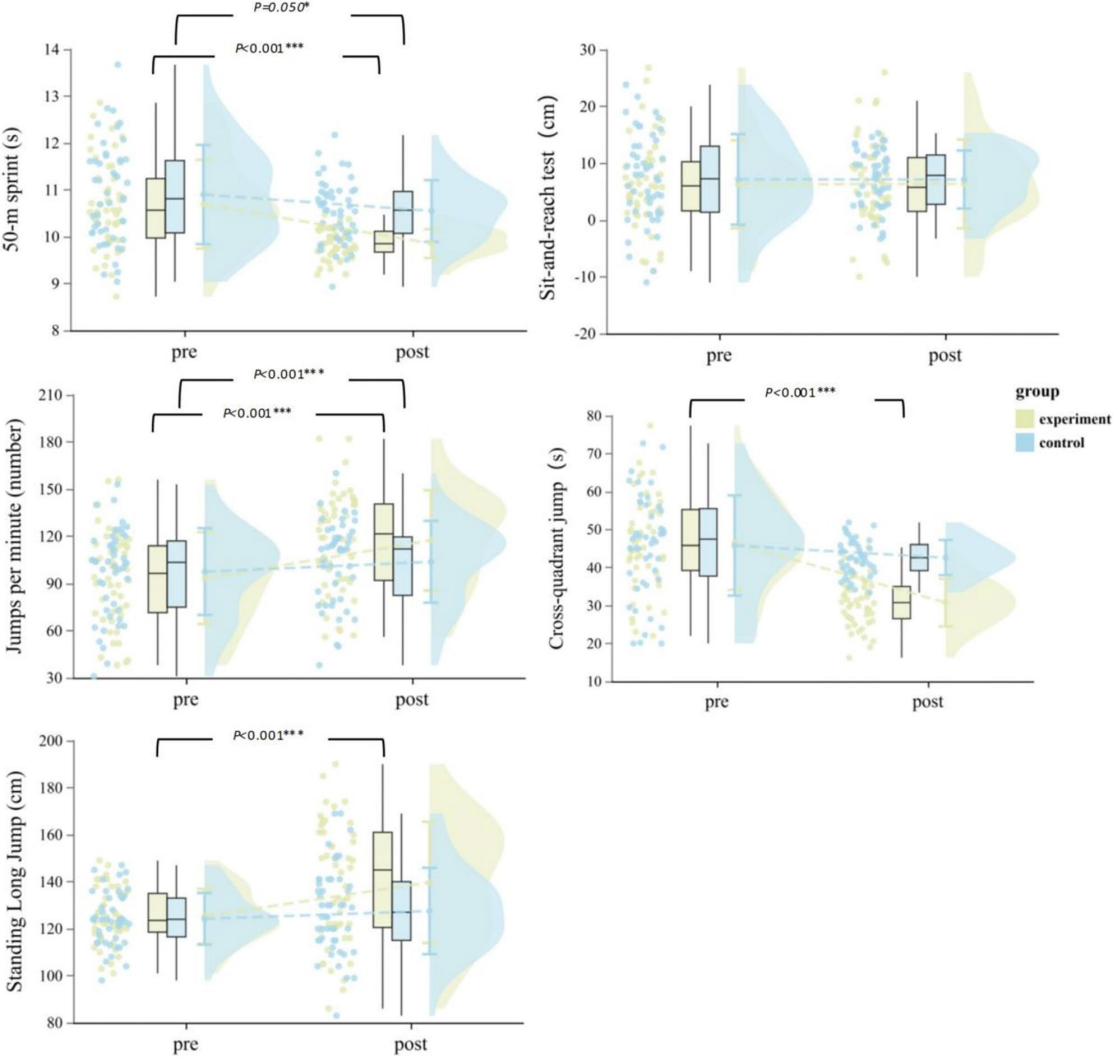
Within-group changes in physical fitness (pre vs. post). Experimental group: yellow, Control group: blue. Only significant within-group changes are marked. ^*^p ≤ 0.05, ^***^p < 0.001

CEPA produced superior improvements across multiple fitness domains (Table 4). Speed(50-m sprint) showed significant time ×group interaction (F(1,92) = 4.59, *p* = 0.035, η²_*p*_ = 0.048, 95% CI [0.00, 0.15]), improving 7.84% in CEPA (*p* < 0.001) versus 3.21% in controls (*p* = 0.050). The gender interaction (F(1,92) = 4.40, *p* = 0.039) revealed girls in CEPA achieved 12.46% improvement (*p* < 0.001) compared to boys’ 3.60% (*p* = 0.124). Endurance(jumps per minute) demonstrated the largest effect (F(1,92) = 154.89, *p* < 0.001, η²_*p*_ = 0.627, 95% CI [0.50, 0.71]), with CEPA improving 25.79% (*p* < 0.001) versus 6.38% in controls (*p* < 0.001). Explosive power(standing long jump) showed a significant time × group interaction (F(1,92) = 4.02, *p* = 0.048, η²_*p*_ = 0.041, 95% CI [0.00, 0.14]), improved significantly in CEPA (11.51%, *p* < 0.001) with no significant change in controls (2.67%, *p* = 0.384). Agility(cross-quadrant jump) showed substantial group differences (F(1,92) = 20.35, *p* < 0.001, η²_*p*_ = 0.181, 95% CI [0.06, 0.31]), improving 33.84% in CEPA (*p* < 0.001) versus 6.94% in controls (*p* = 0.115). Neither intervention affected flexibility(sit-and-reach test) (time × group: *p* = 0.838, η²_*p*_ < 0.001). Complete statistics are provided in Table 3; Fig. 5.

## Discussion

### The effect of CEPA on school-age children’s executive function

Our first hypothesis posited that CEPA would significantly improve executive function in school-aged children. The results supported this prediction, with the CEPA group demonstrating a 15.66% reduction in composite EF score compared to only 2% in TPEC group. The large effect size (η²_*p*_ = 0.634) suggests a robust intervention effect, though this magnitude warrants careful interpretation given our moderate sample size and the behavioral nature of RT-based indices.

Importantly, task-level analyses (Supplementary Table S2) showed that interference cost and switch cost, indices that inherently control for baseline processing speed, were both reduced in the CEPA group, with significant time × group interactions observed across all three EF components. This pattern, together with maintained or improved accuracy, indicates executive-specific gains rather than a generalized speed-up effect.These findings align with Schmidt et al. [84], who reported that cognitively engaging physical activity produced greater improvements in executive function than aerobic exercise, and are further supported by meta-analytic evidence demonstrating robust chronic exercise effects on executive functions in youth populations [85].

The mechanisms underlying CEPA’s effectiveness can be understood through Dual-Task training theory [33], where it is posited that managing simultaneous physical and cognitive demands enhances resource allocation beyond single-task activities. Our intervention systematically incorporated activities requiring concurrent processing of visual/auditory cues while executing complex motor sequences. For instance, activities combining directional movement decisions with rapid response requirements created cognitive-motor interference that prevents reliance on automatic movement patterns. Such tasks demand continuous engagement of multiple executive processes including working memory, inhibition, and cognitive flexibility [2]. This dual activation of prefrontal and motor regions may create synergistic neuroplastic effects exceeding those of single-domain interventions [86, 87].

The contrast between our findings and Egger et al.‘s [44] provides important insights into intervention design. While Egger reported decreased performance following acute cognitively demanding exercise, their single 20-minute session likely captured transient cognitive fatigue from excessive dual-task demands. Our 11-week progressive protocol, however, allowed gradual adaptation to increasing cognitive-motor challenges. This comparison underscores that effective CEPA requires not merely concurrent demands, but carefully calibrated progression that maintains optimal challenge without overwhelming processing capacity. The substantial improvements observed suggest that when appropriately designed, dual-task training can produce meaningful enhancements in executive function among school-aged children.

### The effect of CEPA on school-age children’s mental health

Our second hypothesis was that CEPA would enhance resilience and emotion regulation. The results partially supported this prediction, revealing distinct patterns across different psychological measures. While overall mental health scores (MHT) remained unchanged in both groups, resilience improved significantly by 10.72% in the CEPA group with no change in TPEC group. Similarly, emotion regulation showed greater improvement in the CEPA group (15.69%) compared to TPEC group (5.71%), demonstrating selective benefits for specific psychological capacities.

These selective improvements likely reflect the specific demands of CEPA activities. Unlike TPEC that focuses primarily on motor skills, CEPA requires children to manage multiple challenges simultaneously: processing cognitive information, coordinating with teammates, and adapting to changing rules. These experiences provide repeated opportunities to practice emotional control and develop coping strategies in supportive contexts [88, 89]. The social nature of team-based activities further enhances these benefits, as children learned to regulate emotions while managing both success and failure with peers [90]. Physiologically, physical activity combined with cognitive challenges may enhance neurotransmitter systems involved in mood regulation, with research suggesting that exercise intensity is a critical factor influencing mental health outcomes [91]. Both acute coordinative exercise and chronic exercise training modulate stress-related hormones in children and adolescents [92–94], and exercise-induced hormonal changes may serve as moderators for enhanced cognition in preadolescent populations [95]. Particularly, successful engagement in cognitively demanding tasks has been shown to trigger greater release of pleasure-associated neurotransmitters [96–98].

The contrasting results between specific psychological skills and overall mental health merit consideration. Resilience and emotion regulation are skills that can be directly practiced and strengthened through structured activities, potentially explaining their improvement within our 11-week timeframe. In contrast, the MHT’s comprehensive assessment of mental health status may be less sensitive to short-term interventions than specific skill measures [99]. This pattern is consistent with meta-analytic findings showing that school-based physical activity interventions typically produce stronger effects on specific psychological skills than on global mental health indicators [100]. These findings suggest that CEPA effectively targets trainable psychological capacities, while broader mental health changes may require longer intervention periods (such as 6-month or full academic year programs) or complementary evidence-based approaches (such as psychological counseling or family-centered interventions).

### The effect of CEPA on school-age children’s physical fitness

Hypothesis 3 predicted that CEPA would improve multiple physical fitness indicators. The results strongly supported this hypothesis across most domains. The CEPA group demonstrated superior improvements compared to TPEC group in speed (7.84% vs 3.21% reduction in 50-m sprint time), endurance (25.79% vs 6.38% increase in rope jumping), explosive power (11.51% vs 2.67% increase in standing long jump), and agility (33.84% vs 6.94% improvement in cross-quadrant jump). Only flexibility, measured by the sit-and-reach test, showed no significant improvement in either group.

These comprehensive fitness improvements likely result from CEPA’s unique movement demands. Unlike TPEC that often emphasizes repetitive skill practice, CEPA requires diverse movement patterns integrated with cognitive challenges. This integration necessitates continuous neuromuscular adaptation as children cannot rely on automated movement patterns [35, 84]. The cognitive demands of processing external cues while executing complex movements may enhance motor learning through increased attention to movement quality and real-time error correction [101]. Additionally, the game-based nature of CEPA activities typically elicits higher physical intensity and engagement compared to traditional drill [102], with recent evidence suggesting that acute game-based exercises produce distinct hormonal and cognitive responses that can enhance training adaptations [103].

The observed gender difference in speed improvement (girls: 12.46%, boys: 3.60%) and the lack of flexibility gains warrant specific consideration. The greater speed improvement in girls may reflect baseline differences in physical activity patterns or differential responses to the social and competitive elements of CEPA activities [104]. Regarding flexibility, our intervention prioritized dynamic movements and cognitive challenges over static stretching exercises. This emphasis aligns with our primary objectives but may explain the absence of flexibility improvements. The sit-and-reach test specifically measures hamstring and lower back flexibility, which typically responds to sustained stretching protocols rather than the dynamic movements emphasized in CEPA [105]. This finding suggests that different physical fitness components may require distinct training approaches, with flexibility potentially necessitating dedicated intervention strategies beyond cognitive-motor integration.

### Practical implications

This study provides evidence for integrating cognitive challenges into school-based physical education. The observed improvements in executive function, resilience, emotion regulation, and physical fitness suggest that CEPA may offer advantages over traditional approaches, particularly in resource-limited settings where separate cognitive and physical training programs are not feasible. As a scalable intervention that can be integrated into existing physical education curricula, CEPA represents a cost-effective approach for simultaneously addressing physical and cognitive development.

The differential gender effects observed, particularly in speed development, suggest that CEPA implementation might benefit from adaptive modifications based on student characteristics. While our findings are promising, successful implementation would require consideration of practical factors including teacher training in dual-task activity design, appropriate space and equipment, and methods for maintaining cognitive challenge while ensuring physical activity safety. Educational authorities considering CEPA adoption should pilot test interventions within their specific contexts before broader implementation.

### Strengths and limitations of the study

This study employed a cluster-randomized controlled design with blinded outcome assessors and achieved high participant retention (92.3%). The simultaneous assessment of cognitive, psychological, and physical outcomes provides a comprehensive evaluation of CEPA’s developmental effects.

Several limitations should be acknowledged. First, psychological outcomes relied on self-report measures from 9-year-old children, whose introspective capacity is still developing. Future studies would benefit from incorporating teacher observations, parent ratings, and physiological indicators to enhance measurement validity. Second, despite blinding procedures and continuous monitoring, the use of a single instructor for both groups means that residual expectancy effects cannot be entirely ruled out. Third, the 11-week intervention period precludes conclusions about long-term sustainability. Fourth, our composite EF score is an RT-based behavioral index; future research incorporating neuroimaging measures would strengthen mechanistic interpretations. Fifth, only two clusters were available (one class per condition). Although baseline ICCs were negligible (range: 0–0.001; design effects: 1.00–1.047), standard errors may be underestimated, and effect sizes should be interpreted as within-study effects rather than generalizable population estimates. Finally, this study was retrospectively registered due to administrative processing delays.

## Conclusion

Within the present sample, CEPA produced significantly greater improvements than the TPEC in enhancing EF, resilience, emotion regulation, and several components of physical fitness—particularly agility, coordination, and endurance. Notably, CEPA showed particularly strong effects on EF and emotion regulation. Given that primary school years represent a critical developmental window for executive and socio-emotional functioning, this intervention approach holds considerable potential for broader application within this age range.

Additionally, while no significant gender differences were observed in cognitive and psychological outcomes, CEPA appeared to yield greater gains in physical performance among girls, particularly in speed development, suggesting possible gender-related variation in physical responses to the intervention. Although the program did not significantly improve overall mental health or flexibility, its multi-dimensional benefits underscore CEPA’s value for integration into school-based physical education.

Overall, CEPA not only offers an effective pathway for supporting individual development but also provides preliminary empirical evidence for implementing sustainable, low-cost public health strategies in school settings. Future curriculum design should consider balancing targeted intervention dimensions and addressing individual differences to support more integrated educational models that foster the coordinated development of children’s physical and psychological well-being.

## Data Availability

The anonymized dataset used for analysis will be made available from the corresponding author upon reasonable request.
